# Primary Mucoepidermoid Carcinoma Arising from Ectopic Salivary Tissue within an Intraparotid Lymph Node

**DOI:** 10.1155/2015/879137

**Published:** 2015-11-30

**Authors:** Fatemah Faras, Fawaz Abo-Alhassan, Jassem Bastaki, Mutlaq K. Al-Sihan

**Affiliations:** ^1^Department of ENT, Zain and Al-Sabah Hospitals, Ministry of Health, 40188 Kuwait City, Kuwait; ^2^Department of Surgery, Al-Adan Hospital, Ministry of Health, Kuwait City, Kuwait; ^3^Department of Pathology, Sabah Hospital and Kuwait Cancer Control Center, Ministry of Health, Kuwait City, Kuwait

## Abstract

Ectopic salivary tissue is commonly found in intraparotid and periparotid lymph nodes. Warthin tumor is the most common tumor arising in ectopic salivary gland tissue and in intraparotid lymph nodes. Although rare, neoplastic transformation of the ectopic salivary tissues is conceivable and other types of salivary gland neoplasms arising in intraparotid lymph nodes have been reported. Herein we report a rare case of a 32-year-old Kuwaiti male who presented with a mass in the right parotid gland. A preoperative fine needle aspiration suggested Warthin tumor. The patient underwent a superficial parotidectomy. The specimen showed a mass within the parotid parenchyma abutting the deep margin. Hematoxylin and Eosin stained sections of the lesion showed solid islands and cysts composed of epidermoid cells, mucus cells, and intermixed smaller “intermediate” cells within an intraparotid lymph node. The tumor was seen infiltrating the parotid parenchyma at the deep margin. Metastasis from distant sites was ruled out clinically, and the diagnosis rendered was MEC, low-grade, arising from ectopic salivary tissue in an intraparotid lymph node. Such cases are extremely rare and the presence of malignancies within lymph nodes may pose a diagnostic pitfall, which can affect patient management.

## 1. Introduction

Ectopic salivary gland tissue can be found in many sites including the soft tissue of the neck, the middle ear, the mastoid, and pituitary region [1, 2]. Of particular clinical relevance, ectopic salivary tissue can even be found in intraparotid, paraparotid, and upper cervical lymph nodes [3, 4]. Such salivary tissue may give rise to many neoplasms. In fact, Warthin tumor is the most common tumor arising from ectopic salivary gland tissue within intraparotid lymph nodes [1, 2].

Herein, an extremely rare case of MEC arising primarily within an intraparotid lymph node is presented. To our knowledge, and to date, there has been only one similar reported case in the English literature.

## 2. Case Presentation

A 32-year-old nonsmoker Kuwaiti male was referred to the otolaryngology clinic with a mass on the right side of the face. The lesion was found incidentally on a magnetic resonance imaging (MRI) requested by the patient's neurologist as a follow-up for epilepsy, for which the patient was taking phenytoin.

On local examination a 2.0 × 1.0 cm, nontender mass was located in the right preauricular region. The mass was firm and mobile, with unremarkable overlying skin. A comprehensive physical examination revealed no similar masses or palpable lymph nodes. The facial nerve was functional.

**Figure 1:**
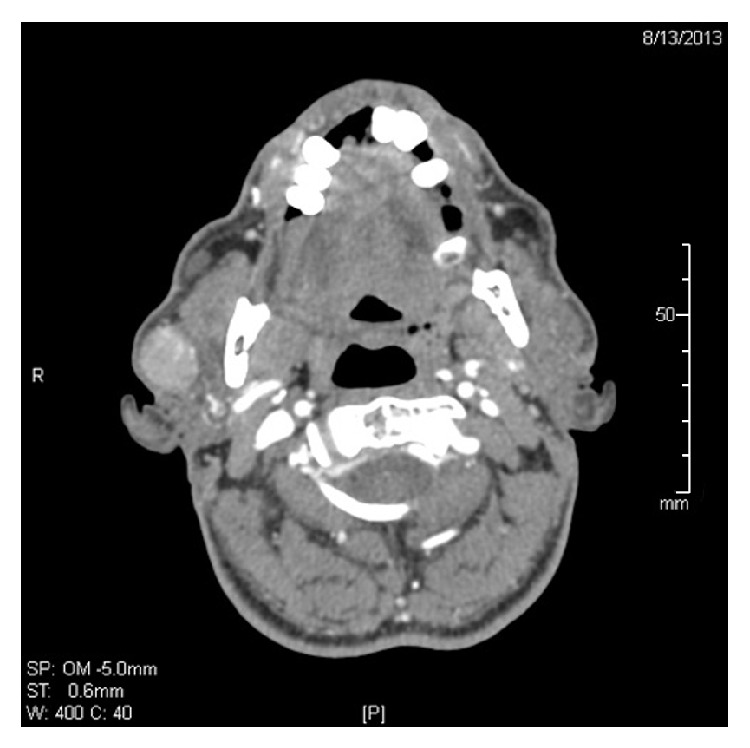
CT neck axial cut, postcontrast, soft tissue window: well-defined oval-shaped mass involving the lobe of the right parotid gland. The lesion shows an increase in density after IV contrast media injection.

Fine needle aspiration cytology (FNAC) was performed and suggested Warthin tumor. The patient underwent a contrast enhanced Computed Tomography (CT) to determine the extent of the lesion. The CT showed a well-defined lesion epicentered in the superficial lobe of the right parotid gland (Figure 1). An elective right superficial parotidectomy was advised and performed by the head and neck surgeon. The lesion was superficial to the facial nerve and the postoperative period was uneventful with an intact facial nerve.

**Figure 2:**
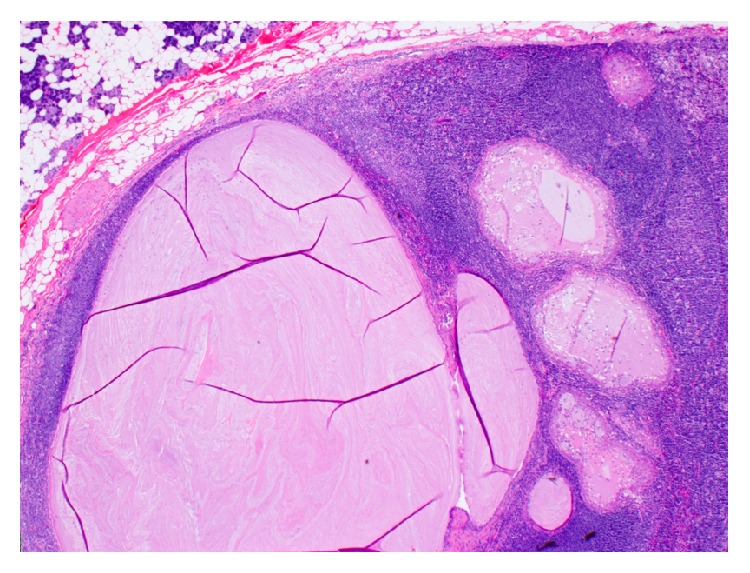
Low magnification of the intraparotid lymph node harboring a cystic neoplasm (20x; H&E).

**Figure 3:**
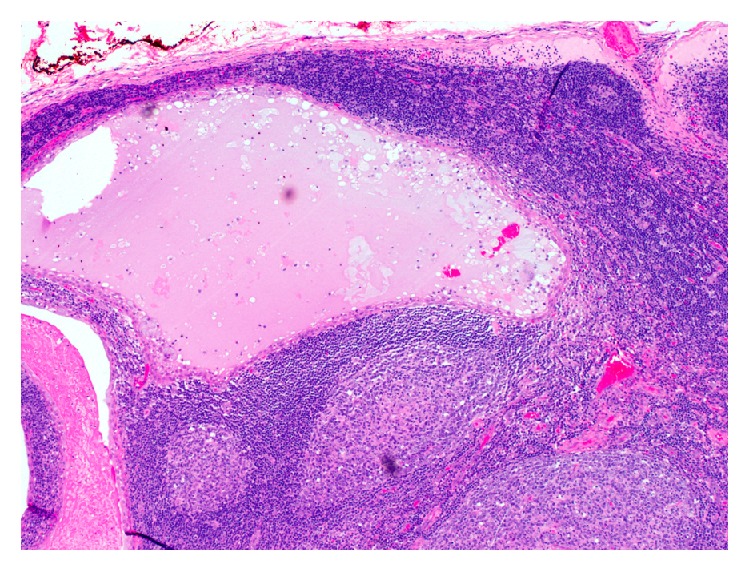
Section of the tumor where the lymph node capsule forms the deep margin focally. Normal lymph node architecture can be seen in this photomicrograph (40x; H&E).

**Figure 4:**
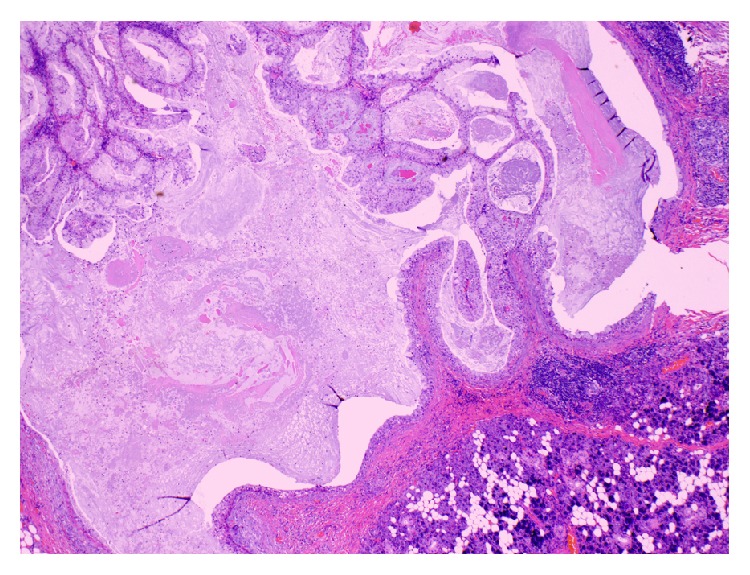
Low magnification photomicrograph showing a focus of the tumor infiltrating outside the lymph node and into the parotid parenchyma (20x; H&E).

**Figure 5:**
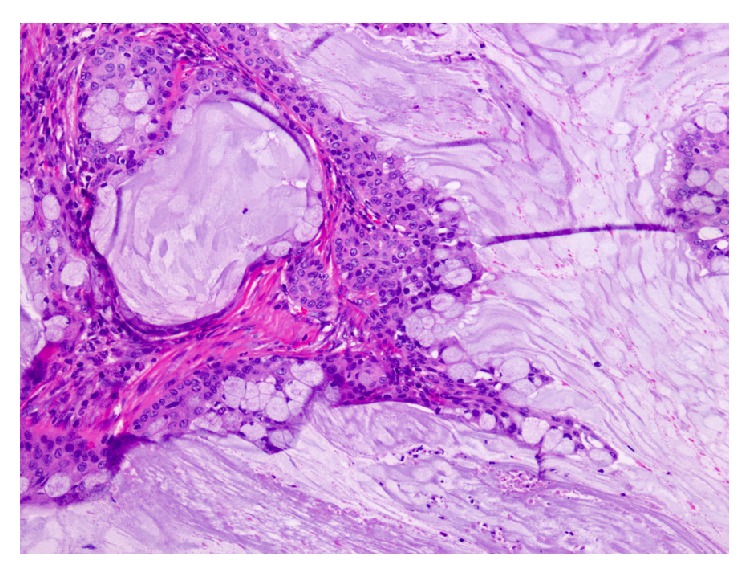
Higher magnification of the tumor shows epidermoid and mucus cells lining the cystic cavities (100x; H&E).

**Figure 6:**
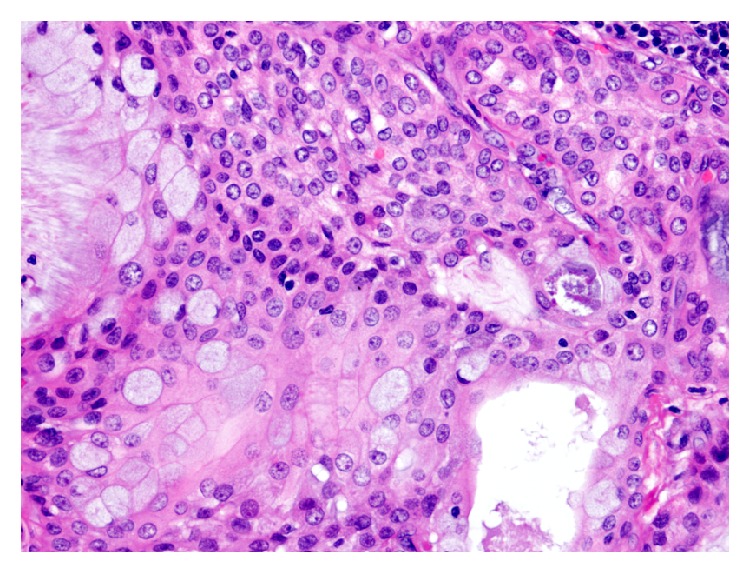
Higher magnification of a more solid focus of the tumor infiltrating outside the lymph node (200x; H&E).

The Hematoxylin and Eosin (H&E) stained sections of the formalin-fixed and paraffin embedded tissue showed a neoplasm composed predominantly of multiple cysts with small solid islands occupying an intraparotid lymph node (Figures 2 and 3) and focally breaking through the capsule and into the parotid parenchyma (Figure 4). At the phenotypic level, the tumor is composed of epidermoid cells with intermixed mucus cells lining the cysts and forming the small islands, most of which are seen outside of the lymph node and infiltrating into the parotid parenchyma (Figures 5 and 6). Angiolymphatic and/or perineural invasion, necrosis, or marked pleomorphism was absent. The mitotic activity was inconspicuous and the tumor was predominantly cystic. No metastasis was identified in any of the other intra and paraparotid lymph nodes. Utilizing the AFIP grading scheme, the tumor was diagnosed as low-grade mucoepidermoid carcinoma (MEC), arising from ectopic salivary tissue within an intraparotid lymph node. The pathologic stage was pT1 N0. Of note, the deep margin was focally and microscopically involved by tumor.

The patient was noncompliant and was lost to follow-up for 18 months. He then returned for his follow-up appointment when an MRI was obtained and he was evaluated clinically. He was confirmed to be disease-free.

## 3. Discussion

In 1985, Smith et al. [1] reported a rare, and rather interesting, case of MEC arising in an intraparotid lymph node. They described a 42-year-old, Hispanic male who presented complaining of a mass in the left parotid region. After superficial parotidectomy, the microscopic examination revealed a low-grade MEC confined entirely to the lymph node, with normal surrounding parotid tissue. The postoperative period was uneventful and one-year follow-up revealed no recurrence of the disease [1].

Heterotopic salivary tissue can be found in many sites, and most importantly intraparotid, periparotid, and upper cervical lymph nodes [1, 3, 4]. Noteworthy, a malignant neoplasm arising from such tissue may provoke a quest to hunt for an unknown primary lesion, especially when it arises within a lymph node. Therefore, both the clinician and the pathologist need to be familiar with such possibility.

Due to the rarity of such occurrences, the pathologist must first consider and rule out a metastatic MEC from a distant site, such as the oral cavity; MEC with tumor associated lymphoid stroma, or Warthin-like stroma; and an infarcted Warthin tumor with squamous metaplasia. In our case, metastasis from another site was ruled out clinically. The presence of normal lymph node architecture, such as a capsule and a subcapsular sinus (Figure 3), ruled out the possibility that this might have been a MEC with tumor associated lymph proliferation (TALP). The lack of a transition from typical squamous islands to oncocytic epithelium and the presence of frank invasion of the tumor outside the lymph node and into the parotid parenchyma aided in excluding an infarcted Warthin tumor.

One might also consider the possibility that the tumor in fact originated from the parotid parenchyma outside the lymph node and then infiltrated the lymph node. However, given that the bulk of the tumor is epicentered in the lymph node with a small tumoral focus outside the lymph node, we felt that such consideration, though possible, is less likely and that it was more likely that the tumor in fact originated inside the lymph node from ectopic salivary tissue. The tumor focus with more solid islands, then, broke through the lymph node capsule and into the parotid parenchyma.

In conclusion, salivary gland neoplasms arising from ectopic salivary tissue are extremely rare and when malignant, a quest to find an unknown primary lesion may be triggered. Therefore, both the pathologist and clinician should be familiar with and consider such presentation only after a metastasis is unequivocally excluded. In cases where a primary lesion is suspected, neck dissection and postoperative radiation therapy should be performed, along with long-term follow-up [5].
